# Chiari I Malformation With Concomitant Nonfunctioning Pituitary and Adrenal Tumors

**DOI:** 10.1210/jcemcr/luae113

**Published:** 2024-06-25

**Authors:** Rachel Zielinski, Aysha Khan, Faryal Sardar Mirza

**Affiliations:** University of Connecticut Health Center: Primary Care Internal Medicine Residency Program, UConn Health, Farmington, CT 06032, USA; University of Connecticut Health Center: Division of Endocrinology and Metabolism, UConn Health, Farmington, CT 06032, USA; University of Connecticut Health Center: Division of Endocrinology and Metabolism, UConn Health, Farmington, CT 06032, USA

**Keywords:** Chiari 1 malformation, nonfunctioning pituitary tumor, nonfunctioning adrenal adenoma, adrenal myelolipoma

## Abstract

Chiari 1 malformation (CM1) is a rare finding that has been described with growth hormone (GH)-secreting pituitary adenomas and with an endothelial PAS domain protein 1 gain-of-function mutation syndrome. We describe the first reported case of a patient diagnosed with CM1 and nonfunctioning pituitary and adrenal incidentalomas. Our case describes a 45-year-old female who was found to have cerebellar tonsillar ectopia consistent with CM1, a pituitary tumor, and bilateral adrenal incidentalomas. She was diagnosed after presenting with 2 weeks of upper extremity weakness and paresthesia. A comprehensive endocrine workup including insulin like growth factor (IGF-1) was normal. She underwent posterior fossa decompression without complication. Pituitary adenectomy was not pursued as there was no evidence of compression of the chiasm or the surrounding structures. In previous case reports it has been proposed that GH-secreting adenomas contribute to CM1 by causing hypertrophy of soft tissue structures in the skull base, overcrowding the posterior fossa. Given that our patient had normal IGF-1 levels, there could be a different underlying mechanism that contributed to the concomitant occurrence of CM1 with the pituitary and adrenal tumors.

## Introduction

The prevalence of adults with symptoms and magnetic resonance imaging (MRI) findings of Chiari 1 malformation (CM1) is 0.01% to 0.04% [1]. It occurs when there is at least 3 to 5 mm of cerebellar ectopy below the foramen magnum and is thought to be genetic due to the underdevelopment of the posterior cranial fossa [2]. There has been a reported correlation between CM1 and growth hormone (GH)-secreting pituitary adenomas, although it is not a definitive mechanism. CM1 has also been described in an endothelial PAS domain protein 1 gain-of-function mutation syndrome with multiple paragangliomas/pheochromocytomas, a somatostatinoma, and polycythemia, also known as Pacak-Zhuang syndrome [3]. There is only 1 case report in the literature describing a nonfunctioning pituitary adenoma with CM1 [4]. In our case, we describe the first reported patient with CM1 and concomitant nonfunctioning pituitary and adrenal tumors.

## Case Presentation

A 45-year-old female with diet-controlled type 2 diabetes, psoriasis, and arterial hypertension presented to the hospital with 2 weeks of bilateral upper extremity weakness, paresthesia, and gait instability. The paresthesia began in the right arm before it spread bilaterally and radiated to both hands. The numbness was the worst in her thumbs and made it difficult for her to perform her activities of daily living including driving. Upon further questioning, she reported an ongoing burning and tingling sensation in her thumb that was originally attributed to carpal tunnel syndrome. She denied visual symptoms, headaches, bowel incontinence, urinary retention, constipation, and saddle anesthesia. A complete neurological exam revealed intact cranial nerves and reduced strength in both upper extremities including shoulder abduction (4/5), elbow flexion (4/5) and extension (3/5), wrist extension (4+/5), and hand grip (3/5). Her lower extremities had some weakness in bilateral hip flexion (4/5) and knee extension (4+/5) and full strength in dorsiflexion, plantar flexion, and great toe extension and flexion. Her sensation to light touch was decreased in the upper and lower extremities in a nondermatomal distribution. She was hyper-reflexive bilaterally in her upper and lower extremities. Hoffman's sign and Babinski sign were positive bilaterally.

## Diagnostic Assessment

**Figure 1.**
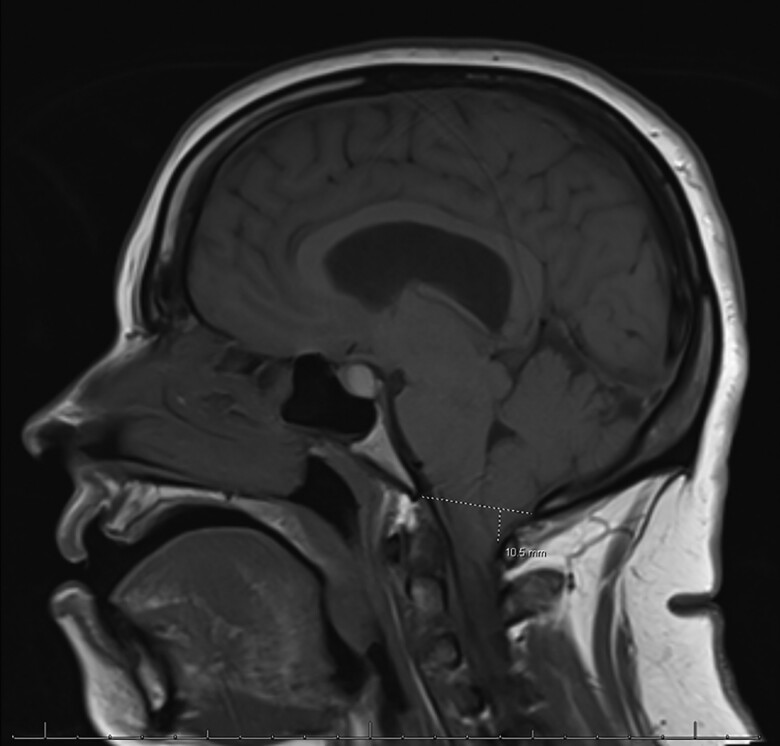
Magnetic resonance imaging brain with and without contrast; T1 sagittal image showing the cerebellar tonsils extending 10.5 mm inferior to the plane of foramen magnum compatible with Chiari I malformation.

**Figure 2.**
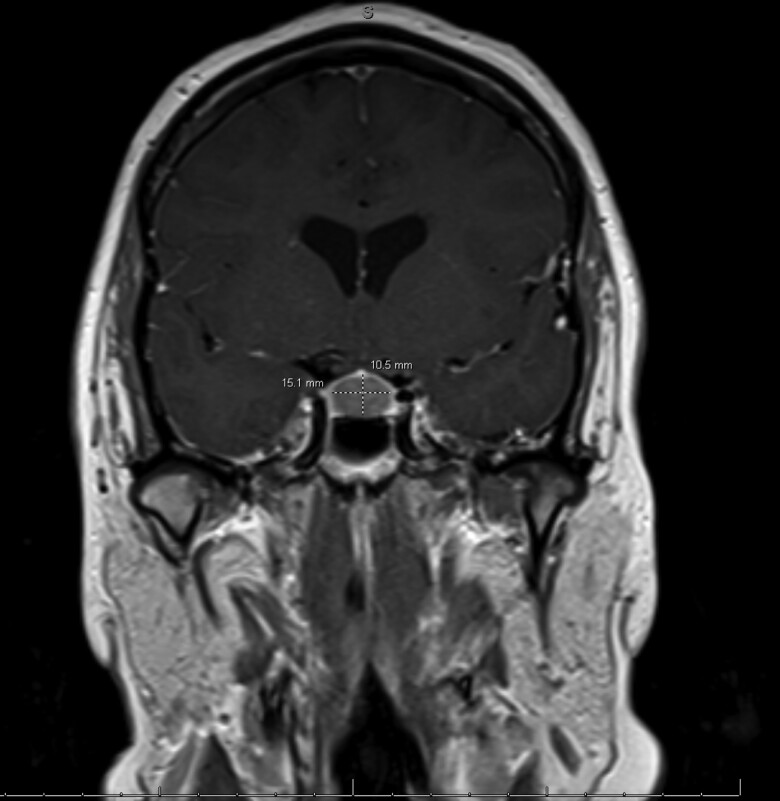
Magnetic resonance imaging brain with without contrast; T1 coronal image showing diffuse enlargement of the pituitary gland: 15.1 mm transverse × 10.5 mm craniocaudad.

**Figure 3.**
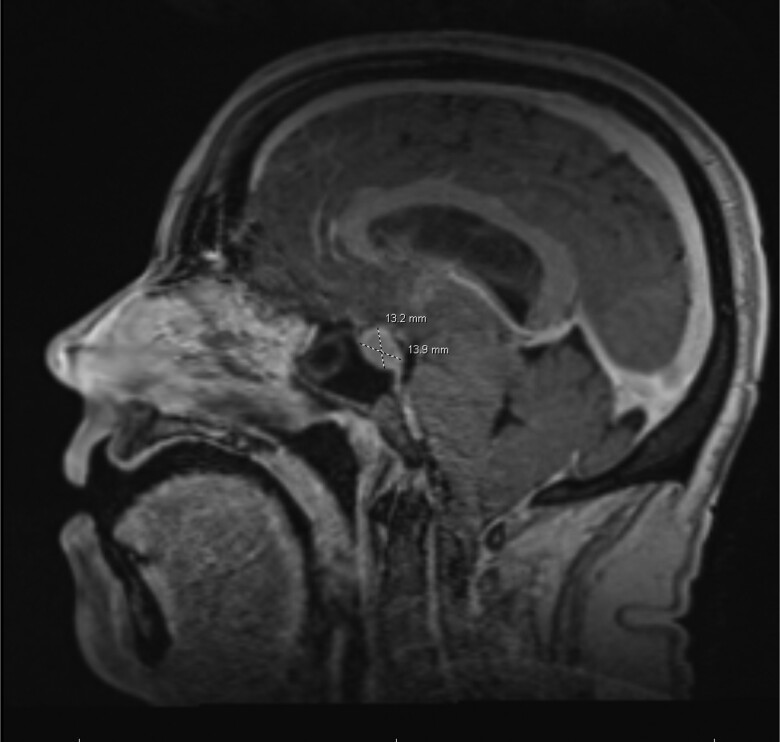
Magnetic resonance imaging brain with without contrast; T1 sagittal image showing the pituitary tumor 13.9 mm anteroposterior.

An MRI of the cervical spine demonstrated cerebellar tonsillar ectopia extending 10.5 mm inferior to the foramen magnum consistent with CM1 and a cervicothoracic syringomyelia (Fig. 1). MRI brain showed diffuse pituitary gland enlargement (15.1 × 13.9 × 10.5 mm) bulging into the suprasellar cistern and minimally abutting the inferior aspect of the optic chiasm, representing either hyperplasia or a macroadenoma (Fig. 2). The lesion was nonenhancing, appeared hyperintense on T1 (Fig. 3), and contained a hypointense foci on T2. MRI lumbar spine revealed a left adrenal gland nodule measuring 15 × 20 mm. Endocrine hormonal evaluation showed normal prolactin of 12.39 ng/mL (12.39 µg/L) (reference range 2.64-13.13 ng/mL, 2.64-13.13 µg/L), FSH of 8.20 mIU/mL (8.20 IU/L) (reference range cycle dependent) and LH of 14.15 mIU/mL (14.15 IU/L) (reference range cycle dependent), estradiol of 295 pg/mL (1083 pmol/L) (reference range cycle dependent), TSH of 0.50 µIU/mL (0.50 mIU/mL) (reference range 0.35-4.94 µIU/mL; 0.35-4.94 mIU/mL), free T4 of 1.04 ng/dL (13.38 pmol/L) (reference range 0.5-1.39 ng/dL; 6.44-17.89 pmol/L), and IGF-I level of 134 ng/mL (17.5 nmol/L) (reference range 66-249 ng/mL; 8.63-32.6 nmol/L), with an age- and sex-adjusted Z-score of −0.1 below the population mean. Plasma aldosterone level was elevated at 57.1 ng/dL (1584 pmol/L) (reference range <16 ng/dL; < 444 pmol/L) in supine position with unsuppressed plasma renin activity at 12.1 ng/mL/hour (12.1 mcg/L/hour) ruling out primary aldosteronism. Pheochromocytoma was ruled out based on normal plasma metanephrine and normetanephrine levels. Dehydroepiandrosterone sulfate was suppressed at 10 µg/dL (0.27 µmol/L) (reference range 35-256 µg/dL; 0.95-6.95 µmol/L). The patient's morning cortisol level after a 1 mg overnight dexamethasone suppression test was <1 µg/dL (<27.6 nmol/L), excluding Cushing syndrome and disease.

## Treatment

The patient was initiated on dexamethasone in anticipation of surgery with a 10 mg intravenous bolus then 4 mg every 6 hours. A random cortisol level prior to starting steroids was 7.3 µg/dL (201 nmol/L) (reference range 3.0-12.0 µg/dL; 82.8 nmol/L −331 nmol/L). Her quadriparesis and gait instability improved; however, she required ambulation assistance due to significant right-sided weakness. One week after discharge, she underwent a suboccipital craniectomy, C1 laminectomy, coagulation of cerebellar tonsils, and expansile duraplasty for CM1. The surgery went well without complications and the patient was tapered off the dexamethasone. She regained strength in her upper extremities and the numbness subsided with only minimal residual numbness in her right thumb.

## Outcome and Follow-up

A brain MRI 3 months postoperatively showed no significant interval change in the size and morphology of the patient's known pituitary tumor. It was again abutting the optic chiasm, but the infundibulum remained midline. A computed tomography scan with adrenal washout showed a low-density right 10 mm × 5.4 mm adrenal nodule consistent with myelolipoma and a left 17 mm × 24 mm adrenal nodule. The left adrenal nodule was 20 Hounsfield units precontrast. Labs done 1 month postoperatively (and off dexamethasone) including TSH, free T4, ACTH, FSH, LH, estradiol, insulin like growth factor (IGF-1), prolactin, cortisol, and aldosterone were all normal. The patient saw neuro-ophthalmology for full visual field testing. The patient had no visual field defects, signs of optic nerve injury on the optical coherence tomography, or any afferent pupillary defect to indicate urgent surgical need. An 84-gene hereditary cancer panel was negative including CDC73, EGFR, HRAS, MEN1, NF1, NF2, and VHL. There was 1 variance of undetermined significance in the adenomatous polyposis coli gene (c.1483A > G). Endothelial PAS domain protein 1 was not tested.

One year postoperatively, the patient is doing well and set to finish respiratory therapy school. She does continue to have some upper extremity weakness, especially in the right deltoid muscle and in both hands, which has improved with physical therapy and occupational therapy sessions. She also has some right-hand burning and paresthesia, which are tolerable on gabapentin.

## Discussion

We present the first case of CM1, a nonfunctioning pituitary tumor, and a nonfunctioning adrenal adenoma incidentally diagnosed simultaneously. While CM1 is a rare finding, pituitary adenomas are relatively common, occurring in 1 in 86.5 to 1 in 2688 patients, and have also been reported in 10% of brain MRIs [2]. In cases of concomitant occurrence of CM1 and acromegaly, it has been proposed that oversecretion of GH causes hypertrophy of connective and ligamentous tissue in the skull base and of the cranio-cervical canal, thus reducing the volume of the posterior fossa and causing tonsillar descent and abnormal cerebrospinal spinal fluid circulation. There have been 9 cases published linking CM1 and acromegaly [3, 5‐8]. Two of them documented reduction of the cerebellar tonsils in a patient with CM1 after treating their acromegaly with transsphenoidal adenectomy [5, 6]. Despite these cases linking GH surplus and CM1, no pathophysiologic mechanism to link the 2 pathologies has been proven. Given that this is the second case to report CM1 occurring with a nonfunctioning pituitary tumor, perhaps GH is not the contributing factor to the CM1 in all of the cases. In the first case to report both pathologies, the patient underwent pituitary adenoma resection prior to the posterior fossa decompression due to the large size of the adenoma and extension into the cavernous sinus. Five years postoperatively, the patient had recovered fully with no neurologic symptoms. Immunohistochemical staining of the adenoma specimen was negative for GH, raising the question of a different hormone or molecule altogether contributing to the CM1 [2].

Our patient had CM1, a nonfunctioning pituitary tumor, and normal GH and IGF-1 levels, raising the possibility of another underlying mechanism, perhaps a genetic predisposition, contributing to the concomitant occurrence of CM1 with pituitary and adrenal tumors and an adrenal myelolipoma. Alternatively, the occurrence could be pure coincidence.

Our patient elected not to have her pituitary tumor resected and currently is being monitored with regular hormonal testing and brain MRIs.

## Learning Points

- Multiple case reports have proposed a relationship between acromegaly and CM1. Our case is the second to describe CM1 and a nonfunctioning pituitary tumor occurring simultaneously.
- Although rare, it is possible to have CM1, a nonfunctioning pituitary tumor, an adrenal adenoma, and an adrenal myelolipoma diagnosed incidentally simultaneously.
- Management requires a multidisciplinary approach with neurosurgery, endocrinology, and neurology input.
- Further investigation and more data are required to determine if there is a relationship between CM1, nonfunctioning pituitary tumors, adrenal adenomas, and adrenal myelolipomas.

## Data Availability

Data sharing is not applicable to this article as no datasets were generated or analyzed during the current study.
